# PERSONAL FACTORS AFFECTING OLDER ADULTS’ INTERPRETATION OF STRESS: MODERATING ROLE OF DIFFERENT LEISURE TYPES

**DOI:** 10.1093/geroni/igad104.3037

**Published:** 2023-12-21

**Authors:** Jaesung An, Soonhyung Kwon

**Affiliations:** California State University East Bay, Hayward, California, United States; Rutgers, the State University of New Jersey, New Brunswick, New Jersey, United States

## Abstract

Holistic stress model (Nelson & Simmons, 2003; 2011) offers the most updated framework to understand how individuals’ differences in characteristics (e.g., locus of control, optimism, hardiness) determines their interpretation of the given stressor, whether as eustress or distress. However, dearth of literature exists to further validate this framework, and barely any study has been conducted among older adult population. While daily leisure engagement is one of the most valued activities among older adults, which helps them navigate healthy aging process (An et al., 2022), it also has positive relationships with older adults’ sense of control, positive emotions, and resilience (Son et al., 2021). Therefore, the purpose of this study was to examine whether individual differences (sense of control, optimism, social support, spiritual belief) have effects on how older adults respond to given stressor and to investigate whether there is a moderating effect of participating in different types of leisure activities. This study used the Health and Retirement Study (HRS) data and study participants (n=3,038) aged 64.52 years old on average. Multiple linear regression was used for analyses using SPSS. Findings indicated that higher social support, optimism, and sense of control were significantly associated with lower response to chronic stress, whereas higher spiritual belief was related to higher response to chronic stress. In terms of specific moderator effects, physical leisure was a significant moderator between social support, optimism, sense of control, and spiritual belief and response to chronic stress. Further discussion, implication, and future recommendations will be presented.

